# Patient-reported outcomes in patients with peristomal pyoderma gangrenosum: A single-center prospective study

**DOI:** 10.1016/j.jdcr.2024.10.015

**Published:** 2024-11-05

**Authors:** Tanya Magana, Katherine M. Erickson, Khoa Nguyen, Alex G. Ortega-Loayza

**Affiliations:** a Department of Internal Medicine, Oregon Health and Science University, Portland, Oregon; b Department of Dermatology, New York Medical College, Valhalla, New York; c Department of Dermatology, Oregon Health and Science University, Portland, Oregon

**Keywords:** inflammatory bowel disease, peristomal pyoderma gangrenosum, quality of life, stoma, wound characteristics

Among patients with inflammatory bowel disease who undergo stoma surgery, 2.0% to 4.3% develop peristomal pyoderma gangrenosum (PPG), causing inflammatory ulcerations surrounding the stoma.^1^, ^2^, ^3^, ^4^ This has drastic impacts on quality of life that have not yet been described. Thus, we aimed to quantify the impact of having PPG in our prospective cohort of patients in the pyoderma gangrenosum (PG) pilot registry at Oregon Health & Science University. The data collection between June 2021 and January 2024 included ulcer and stoma characteristics, pain (numerical rating scale [NRS] scale), patient global assessment, Wound-QOL-14 (WQ14) and Skindex-Mini (SDM) scores, and prescription pain medication use. We included patients with a diagnosis of PPG who had 2 or more clinic appointments that were at least 6 weeks apart; median follow-up time was 22 months. We performed two-tailed t-tests to compare means and evaluated correlations with Spearman’s coefficient (r_s_).

We identified 10 patients with PPG whose characteristics are summarized in Table I. Seven patients had Crohn’s disease, 1 had ulcerative colitis, and 2 had malignancy without inflammatory bowel disease. The median baseline ulcer size was 3.1 cm with the most common depth to the dermis. The median baseline values for ulcer age was 3.5 months, pain was 4/10 on the NRS scale, and patient global assessment was 3/4 (moderate severity). The mean global WQ14 was significantly different (*P* = .018) between patients with ulcers to the dermis (1.4) compared to the subcutis (2.1). WQ14 correlated moderately with depth (r_s_ = 0.34) but strongly with ulcer size (r_s_ = 0.58). Seven patients took pain medications and had baseline mean wound age, size, and pain scores of 7 months, 11.9 cm, and 5.3 compared to patients not taking pain medications, which were 1.7 months, 3.9 cm, and 3.3, though these differences were not statistically significant (*P* = .28, .58, and 0.32, respectively).

**Table I:** Demographics, baseline clinical characteristics, and quality of life scores for patients with peristomal pyoderma gangrenosum (PPG) (*n* = 10)

	No. (%)
Women	10 (100)
Age, mean (y)	51.6
Race	
White	9 (90)
Prefer not to say	1 (10)
Stoma type	
Ileostomy	9 (90)
Urostomy	1 (10)
Stoma age at first visit (y)	
<1	5 (50)
1-2	1 (10)
3-10	1 (10)
15-20	3 (30)
Prescribed oral pain medication	
Yes	7 (70)
No	3 (30)
Baseline quality of life (mean)	
Wound-QoL global score	1.37
Wound-QoL body subscore	1.28
Wound-QoL psych subscore	1.75
Wound-QoL life subscore	1.29
Skindex-Mini global score	2.9
Skindex-Mini symptom subscore	3.2
Skindex-Mini emotion subscore	2.8
Skindex-Mini function subscore	2.8
PG-associated comorbidities	
Crohn’s disease	7 (70)
Ulcerative colitis	1 (10)
Malignancy	2 (20)
Pain-associated comorbidities	
Fibromyalgia	3 (30)
Major depressive disorder	3 (30)
Migraine	2 (20)

Patients with PPG seem to present to clinic earlier in their disease course compared to patients with classic PG. Patients with stomas may seek medical care earlier as they are well-acquainted with the health care system and have access to specialized ostomy nurses. Even still, at baseline, patients with PPG rated their skin disease as moderate, which highlights the negative impacts their disease has on their life. Patients with deeper ulcers reported significantly worse quality of life; however, WQ14 correlated more strongly with ulcer size than depth, perhaps due to the additional burden and more restrictive lifestyle associated with covering a large ulcer as opposed to a deep ulcer that is small. At baseline, patients not taking pain medications presented earlier with smaller, shallower, and less painful ulcers compared with patients who were prescribed pain medications, which may suggest that earlier evaluation may reduce the need for analgesics. While the differences between these groups were not significant, this was likely due to the small sample size rather than the magnitude of difference. In conclusion, this study demonstrates that PPG negatively impacts quality of life and pain intensity. Early intervention may reduce the need for analgesia, but further research is needed to confirm these potential benefits.

## Conflicts of interest

Dr Ortega-Loayza reports consultancy/advisory boards disease-relevant honoraria from Genentech, Boehringer-Ingelheim, Bristol Mayer-Squibb and Janssen. Dr Ortega-Loayza also reports research grants from Lilly, Pfizer and Janssen. Dr Ortega-Loayza is also supported by NIAMS NIH R01AR083110. Drs Magana, Erickson, and Nguyen have no conflicts of interest to declare.
